# Dataset of physiological, behavioral, and self-report measures from a group decision-making lab study

**DOI:** 10.1016/j.dib.2022.108630

**Published:** 2022-09-24

**Authors:** Alon Burns, Sebastian Wallot, Yair Berson, Ilanit Gordon

**Affiliations:** a Department of Psychology, Bar-Ilan University, Ramat-Gan, Israel; b Institute of Psychology, Leuphana University of Lüneburg, Lüneburg, Germany; c DeGroote School of Business, McMaster University, Hamilton, ON, Canada

**Keywords:** Group social interactions, Electrodermal activity, Heart rate, Behavioral microanalysis

## Abstract

This paper presents data from a study conducted in twenty groups of three participants each. Data were collected from sixty participants during a lab visit which was video recorded from several angles. Upon arrival to the lab and following informed consent, participants were told that they would be a part of a group decision-making task and were given instructions for a procedure titled “the desert survival task” Lafferty and Pond (1974). Participants were then connected to several electrodes on their upper body and palm for the collection of their electrocardiogram, respiration and electrodermal activity throughout the group task. Participants then performed the task together. The collection of physiological data from all group members was conducted simultaneously and in synchrony with the video recording. The video recordings of the group interactions were later coded by trained psychology students for positive affective behaviors made by participants (smiling and laughing) throughout the group task. Self-report measures (trait anxiety and social phobia) were collected prior to the group task from all participants. This multimodal dataset thus integrates behavioral, self-report, and physiological measures from group members, which are important for understanding group dynamics. These data will allow verification, replications, and additional analyses of the data from new perspectives.

**Specifications Table**

**Table:** 

Subject:	Neuropsychology and physiological psychology
Specific subject area:	This area of research focuses on the physiological mechanisms of behavior using measures of the nervous system
Type of data:	Tables
How the data were acquired:	Physiological data were acquired using MindWare Impedance Cardiograph mobile recorders connected to each individual in the group via electrodes at 500 Hz. (MindWare Technologies, Gahanna, OH). Electrocardiogram data (ECG) were obtained using a modified lead II configuration and the respiratory data for the analysis of IBI were obtained using the standard tetrapolar electrode system for impedance recording [2]. Electrodermal Activity (EDA) was collected using two disposable Ag-AgCl electrodes, placed on the palm of participants’ nondominant hand. EDA and heart rate values were later outputted from the MindWare EDA/HRV analysis software. Upon arrival at the lab, participants filled out questionnaires about their level of anxiety and social phobia, and each group was recorded for later coding of positive affective behavior.
Data format:	Mixed (raw and preprocessed)
Description of data collection:	Participants were contacted via email by the experimenter and they agreed to participate in the study. Upon arrival to the lab, participants provided informed consent and filled up self-report questionnaires. They were told that they would be taking part in a joint decision-making task. The task was explained to the participants who were then connected to electrodes for physiological data collection and performed the desert survival task while being videorecorded.
Data source location:	Data were collected from undergraduate students in the Department of Psychology at Bar-Ilan University, Ramat Gan, Israel.
Data accessibility:	Repository name: Mendeley Data Data identification number: 10.17632/w8h752hbw5.1 Direct URL to data: https://data.mendeley.com/datasets/w8h752hbw5/3
Related research article:	I. Gordon, S. Wallot, Y. Berson, Group‐level physiological synchrony and individual‐level anxiety predict positive affective behaviors during a group decision‐making task. Psychophysiology, 58 (2021). https://www.doi.org/10.1111/psyp.13857

## Value of the Data

• This multimodal dataset includes behavioral, physiological, and self-report measures taken as part of a lab study. This method of integration necessitates a lab facility that allows for such complex data collection with specialized equipment and expertise and is therefore not often accessible to the research community.
• Sharing these data will allow other researchers to use multimodal data for different purposes, such as reanalyzing data via different analysis methods, or examining novel research questions and models, theory-building and meta-analysis.
• Shared datasets, like this one, support data verification and encourage replications [5].
• This dataset will allow researchers to compare different methodologies used to assess physiological synchrony. The original paper examined physiological synchrony between participants using multidimensional recurrence quantification analysis (MdRQA) [6]. However, there are established alternative methods to calculate physiological synchrony, which may result in different or refined outcomes [7].

## 1 Data Description

This dataset includes three types of data: Physiological, self -report survey responses and behavioral data. The survey responses and behavioral data are presented together in a table which includes each participant's demographic information, the duration of positive affective behavior coded from videos (in seconds) and participants’ responses to the STAI and SPIN surveys (Table 1). The surveys are provided as a supplementary file. Several physiological functions were also collected: ECG, cardiological impedance and EDA. These data allowed us to derive Heart Rate (HR), Heart Rate Variability (HRV) and EDA measures. In the data files there is one folder per participant, that contains their pre-processed HRV (Table 2) and EDA (Table 3) data.

**Table 1:** Variable description of survey responses and behavioral data.

Category	Sub-Category	Item name	Type	Description	Source
General	Identification codes	ParticipantID	Integer	Code generated from each participant number	Assigned
		DyadID	Integer	Random code generated from each participant number	Assigned
		GroupID	Integer	Code generated from each group number	Assigned
		SubjectID	Integer	Serial participant number	Assigned
		Group_number	Integer	Serial group number	Assigned

Demographic	Age	Age	Integer	Age in years	Self-report
	Education Years	Education_years	Integer	Number of years in a formal education program	Self-report

Behavioral	Total duration of positive affect	Total_duration_positive	Decimal	Time (in seconds) that participants either smiled or laughed throughout the group task	Coded from video data
	Duration of positive affect in percentage	Percentage_observation _duration_positive	Decimal	Percentage of time from the duration of the group task in which participants either smiled or laughed	

Survey responses	Trait anxiety	STAI1	Integer	I feel calm	Scale of 1-7
		STAI2	Integer	I feel secure	
		STAI3	Integer	I feel tense	
		STAI4	Integer	I feel strained	
		STAI5	Integer	I feel at ease	
		STAI6	Integer	I feel upset	
		STAI7	Integer	I am presently worrying over possible misfortunes	
		STAI8	Integer	I feel satisfied	
		STAI9	Integer	I feel frightened	
		STAI10	Integer	I feel uncomfortable	
		STAI11	Integer	I feel self-confident	
		STAI12	Integer	I feel nervous	
		STAI13	Integer	I feel jittery	
		STAI14	Integer	I feel indecisive	
		STAI15	Integer	I am relaxed	
		STAI16	Integer	I feel content	
		STAI17	Integer	I am worried	
		STAI18	Integer	I feel confused	
		STAI19	Integer	I feel steady	
		STAI20	Integer	I feel pleasant	
		STAI1r	Integer	Reversed score for item 1	
		STAI2r	Integer	Reversed score for item 2	
		STAI5r	Integer	Reversed score for item 5	
		STAI8r	Integer	Reversed score for item 8	
		STAI11r	Integer	Reversed score for item 11	
		STAI15r	Integer	Reversed score for item 15	
		STAI16r	Integer	Reversed score for item 16	
		STAI19r	Integer	Reversed score for item 19	
		STAI20r	Integer	Reversed score for item 20	
	Social phobia	SPIN1	Integer	I am afraid of people in authority	Scale of 0-7
		SPIN2	Integer	I am bothered by blushing in front of people	
		SPIN3	Integer	Parties and social events scare me	
		SPIN4	Integer	I avoid talking to people I do not know	
		SPIN5	Integer	Being criticized scares me a lot	
		SPIN6	Integer	I avoid doing things or speaking to people for fear of embarrassment	
		SPIN7	Integer	Sweating in front of people causes me distress	
		SPIN8	Integer	I avoid going to parties	
		SPIN9	Integer	I avoid activities in which I am the center of attention	
		SPIN10	Integer	Talking to strangers scares me	
		SPIN11	Integer	I avoid having to give speeches	
		SPIN12	Integer	I would do anything to avoid being criticized	
		SPIN13	Integer	Heart palpitations bother me when I am around people	
		SPIN14	Integer	I am afraid of doing things when people might be watching	
		SPIN15	Integer	Being embarrassed or looking stupid are among my worst fears	
		SPIN16	Integer	I avoid speaking to anyone in authority	
		SPIN17	Integer	Trembling or shaking in front of others is distressing to me

**Table 2:** Variable description of HRV data.

Category	Description	Variable	Source
HRV Stats	The outputted data from the HRV analysis module in the MindWare HRV application.	Mean heart rate	Average heart rate in beats per minute
		RSA (Respiratory Sinus Arrhythmia)	Integral of the signal in the high frequency band in ms^2^
		Mean IBI (Inter-Beat Interval)	Average IBI in milliseconds
		# of R's Found	Total number of R peaks
		Respiration Rate	Average breaths per minute
		Respiration Amplitude	Power of the respiration peak frequency in volts
		Respiration Peak Frequency	RSA frequency within the band with the most spectral power in Hz
		Respiration Power	Integral of the RSA in the specified frequency band in ms^2^
		First ECG R Time	Integral of the Resp Power Spectrum series
		SDNN	The standard deviation of N-N intervals
		AVNN	Average N-N interval
		RMSSD	The root mean square of differences between successive N-N intervals
		NN50	The number of N-N intervals which differ by >50ms from the previous interval
		pNN50	Ratio of NN50 in the total time series

IBI Series	Timing between all annotated beats	IBI intervals	Time between all annotated beats in milliseconds

Power Band Stats	The MindWare HRV analysis application uses a Fast Fourier Transform (FFT) to look at specific frequencies of variability by transforming the IBI series from the time domain to the frequency domain	VLF Power	Integral of the signal in the Very Low Frequency band in ms^2^
		VLF Peak Power Frequency	IBI frequency within the Very Low Frequency band with the most power in Hz
		LF Power	Integral of the signal in the Low Frequency band, measured in ms^2^
		LF Peak Power Frequency	IBI frequency within the Low Frequency band with the most power in Hz
		HF/RSA Power	Integral of the signal in the High Frequency band in ms^2^
		HF/RSA Peak Power Frequency	IBI frequency within the High Frequency band with the most power in Hz
		LF/HF Ratio	Ratio of power in the LF and HF/RSA bands

Heart Period Time Series	A frequency-based interpolation of the IBI series	Interpolated IBI intervals	Interpolated IBI series in milliseconds

Respiration Time Series	This measure is derived from the variability in time between heart beats	Respiration Delta	RSA time series in volts

Respiration Power Spectrum	A frequency-based transformation of the RSA	FFT transformed RSA	RSA frequencies with the most power in Hz

**Table 3:** Variable description of EDA data.

Category	Description	Variable	Source
EDA Stats	This tab in the spreadsheet presents the output data of the EDA analysis module in the MindWare EDA application.	Total SCRs	Total number of Skin Conductance Responses
		ER-SCRs	Number of Event Related Skin Conductance Responses
		NS-SCRs	Number of Non-Specific Skin Conductance Responses
		Tonic SCL	Average Skin Conductance Level, excluding SCRs, in microsiemens (uS)
		Mean SC	Average Skin Conductance Level in microsiemens (uS)
		Tonic Period	Total time of waveform, excluding SCRs, in milliseconds

SCR Stats	Skin Conductance Response (SCR) stats from the entire time series	SCR Type	A response is event related (ER) if the difference between the event time and the trough of the response is greater than the ER-SCR latency min and less than or equal to the ER-SCR latency max. A non-specific (NS) skin conductance response does not meet the timing criteria specified by the ER-SCR latency min and ER-SCR latency max
		Event Time	Time of SCR in milliseconds if it is event related
		Event Type	Type of related event if the SCR is event related (Not available)
		Trough Time	Time of the trough associated with each SCR in milliseconds
		Peak Time	Time of the peak associated with each SCR
		HR Time	Half-recovery time for each SCR
		Trough SCL	Amplitude of the trough for each SCR
		Peak SCL	Amplitude of the peak for each SCR
		SCR	Total skin conductance level for each SCR

Interval Stats	Mean skin conductance (SC) for each interval in the time series	Interval Start Time	Interval Start Time
		Interval End Time	Interval End Time
		Mean SC	Mean SC for each interval

## 2 Experimental Design, Materials and Methods

Participants were undergraduate students in psychology (*N* = 60) who took part in the study in exchange for course credit or payment. Students were contacted via email by a research assistant, and a time slot was suggested during which they were to participate in the study. After agreeing to participate in the study, participants were asked to arrive at the lab well hydrated and to avoid caffeinated drinks and nicotine for two hours before the start. It was further explained that the study would include physiological measurements using electrodes which are non-invasive and neither dangerous nor painful. Furthermore, the participants were told that the entire study will be recorded for later analysis, which will only be conducted by members of the research team. Upon arrival to the lab, participants were informed that they would take part in a joint decision-making task in groups of three and they provided informed written consent. Participants then reported certain demographic information (age, gender) and filled in self-report questionnaires for anxiety and social phobia – “STAI” and “SPIN”). Participants were then connected to MindWare mobile recorders (MindWare Technologies, Gahanna, OH). Participants were then asked to sit and relax for five minutes, not moving or talking at all for a physiological baseline period.

Next, participants started the desert survival group task [1]. This task was developed and utilized for examining group dynamics and it was chosen as it allowed for a social group discussion that would give rise to social interactions and be appropriate for multimodal data collection. Participants were told they had survived an airplane accident which left them stranded in the middle of a desert. Each participant was asked to rank 15 items for survival according to importance. Next, participants were asked to discuss their ranking as a group and to reach a mutually acceptable agreement for a group ranking of the 15 items necessary for survival [1].

Positive affective behavior (smiles, laughter) was coded by two independent trained psychology students from 20 videos recorded in the lab during the study. At least 85% interrater reliability was reached for all coders in the 3 videos of each group. Coders annotated each time participants started and stopped smiling or laughing. The durations of time that group members displayed positive affect were calculated from the Noldus Observer XT (Wageningen, the Netherlands) - the event logging behavioral coding software that coders used.

To quantify individuals’ traits, we used two self-report measures (provided as a supplementary file): The state trait anxiety inventory (STAI) [3] and the social phobia inventory (SPIN) [4].

Physiological data were pre-processed by trained graduate students using the MindWare Technologies HRV and EDA applications software (v3.1.4). HRV data were visually examined to ensure the removal of artifacts and ectopic beats. The signals were amplified by a gain of 1000 and filtered with a Hamming windowing function. EDA data were visually examined to identify unusual peaks or drops, which indicate corrupted data. These corrupted sections in the data were replaced using a linear spline interpolation. EDA data were excluded from the final analysis if more than 5% of the participant's data were identified as corrupted. Some data are missing in the dataset for the following reasons: (1) Positive affect could not be coded for three participants as a result of technical issues with the video recordings in one group, (2) EDA data was not properly recorded for thirteen participants because of motion artifacts and technical issues with the MindWare mobile recorders, (3) Six participants did not complete the STAI and SPIN surveys, and (4) Four participants did not provide demographic information of their number of years in formal education programs and age, and one additional participant did not provide their age.

## Ethics Statements

The Institutional Review Board of the Department of Psychology at Bar-Ilan University approved this study and we adhered to their ethical guidelines (12/2017). Informed consent of all participants has been obtained.

## CRediT authorship contribution statement

**Alon Burns:** Writing – review & editing, Data curation. **Sebastian Wallot:** Conceptualization. **Yair Berson:** Conceptualization. **Ilanit Gordon:** Conceptualization, Writing – review & editing, Data curation.

## Declaration of Competing Interest

The authors declare that they have no known competing financial interests or personal relationships that could have appeared to influence the work reported in this paper.

## Data Availability

Dataset of physiological, behavioral, and self-report measures from a group decision-making lab study (Original data) (Mendeley Data).

## Appendix Supplementary materials

Image, application 1
